# Green TPUs from Prepolymer Mixtures Designed by Controlling the Chemical Structure of Flexible Segments

**DOI:** 10.3390/ijms22147438

**Published:** 2021-07-12

**Authors:** Paulina Kasprzyk, Ewa Głowińska, Paulina Parcheta-Szwindowska, Kamila Rohde, Janusz Datta

**Affiliations:** Department of Polymer Technology, Faculty of Chemistry, Gdansk University of Technology, 11/12 Gabriela Narutowicza Street, 80-233 Gdansk, Poland; ewa.glowinska@pg.edu.pl (E.G.); paulina.parcheta@pg.edu.pl (P.P.-S.); kamila.blazek@pg.edu.pl (K.R.)

**Keywords:** thermoplastic polyurethane elastomers, green TPUs, segmented polyurethanes, flexible segments, chemical structure

## Abstract

This study concerns green thermoplastic polyurethanes (TPU) obtained by controlling the chemical structure of flexible segments. Two types of bio-based polyether polyols—poly(trimethylene glycol)s—with average molecular weights ca. 1000 and 2700 Da were used (PO3G1000 and PO3G2700, respectively). TPUs were prepared via a two-step method. Hard segments consisted of 4,4′-diphenylmethane diisocyanates and the bio-based 1,4-butanodiol (used as a chain extender and used to control the [NCO]/[OH] molar ratio). The impacts of the structure of flexible segments, the amount of each type of prepolymer, and the [NCO]/[OH] molar ratio on the chemical structure and selected properties of the TPUs were verified. By regulating the number of flexible segments of a given type, different selected properties of TPU materials were obtained. Thermal analysis confirmed the high thermal stability of the prepared materials and revealed that TPUs based on a higher amount of prepolymer synthesized from PO3G2700 have a tendency for cold crystallization. An increase in the amount of PO3G1000 at the flexible segments caused an increase in the tensile strength and decrease in the elongation at break. Melt flow index results demonstrated that the increase in the amount of prepolymer based on PO3G1000 resulted in TPUs favorable in terms of machining.

## 1. Introduction

Polyurethanes constitute some of the most versatile materials, of which all kinds, such as cast polyurethanes, foams, coatings, and adhesives, have many practical applications in everyday life. Their selected properties, purpose, form, texture, and other features can be modified by manipulating the quality and amount of ingredients. One type of polyurethane material, which has been gaining interest in recent years, is thermoplastic polyurethane elastomers—TPUs [1,2,3,4]. These materials are characterized by a segmented structure that is formed by two types of segments: flexible, consisting of polyols (polyester or polyether macrodiols), and hard, composed of products of the reaction of isocyanates and chain extenders [5,6]. Long flexible chains are responsible for the elastomeric properties of TPUs. During tensile deformation, the flexible segments uncoil and allow the polymer to stretch several times its original length. The entropic energy stored in the soft segments provides the driving energy to return the polymer to its original shape. The hard segments behave as pinning points that “remember” the original shape [7]. The chemical structure and the flexible and hard segment content are also responsible for the physicochemical and thermal properties, purpose, form, texture, and many other features of elastomeric polyurethane materials.

In recent decades, there has been a visible increasing interest in sustainability in the chemical industry. Currently, the trend related to the use of bio-based resources in chemical synthesis is being strongly developed by scientists from the research sector and R&D specialists from chemical industries. This trend is also clearly visible in the polyurethane industry. Nowadays, selected chemicals of natural origin are commonly available and can be used as bio-based monomers for the preparation of polyurethane materials [1,8,9,10,11,12,13]. For instance, there are commercially available monomers for the synthesis of such bio-based polyols as succinic acid, sebacic acid, 2,5-furanodicarboxylic, ethylene glycol, 1,3-propanodiol, and 1,4-butanodiol. In the research literature, we can find out information about the use of such bio-based polyols as poly(trimethylene succinate)s [4], poly(trimethylene glycol)s [2,14], and poly(propylene carbonate) [15] for the preparation of TPUs. Moreover, there are a lot of work that describe results of using polyols based on vegetable oils such as rapeseed oil [16], seed oil [17], and soybean oil [18,19]. Nevertheless, bio-based polyols are not the only bio-based ingredients of polyurethane materials. Currently, bio-based isocyanates for PU synthesis are also being developed [20].

Based on the comparison between a bio-based polyether polyol (poly(trimethylene glycol) PO3G) and its petrochemical counterparts (poly(tetramethylene-ether)glycol PTMEG), it was verified that the bio-based polyol has a significantly lower environmental footprint, saving 40% in nonrenewable energy consumption and reducing greenhouse gas emissions by 42%, as proven by an ISO 14000-compliant life cycle analysis [21].

Researchers developing the use of bio-based monomers in the synthesis of polyurethanes have investigated the effect of bio-based soft and hard segments on selected properties of bio-PUs. For instance, Pattamaprom, Rwei, and co-workers [22] investigated the impact of different average molecular weights of bio-based soft segments on selected properties of green thermoplastic polyurethanes with temperature-sensitive shape memory behaviors. The authors prepared bio-TPU materials with the use of the one-shot solvent-free polymerization of bio-poly(trimethylene succinate) glycol (PPS) with various molecular weights (ca. 1000, 2000, 3000, and 4000 Da), 1,4-butanediol (BDO), and 4,4′-methylene diphenyl diisocyanate (MDI). They investigated the effect of the molecular weight of the soft segment on the mechanical, thermomechanical, and shape memory properties of these TPUs. The obtained results allowed the confirmation that the soft segment with high molecular weight (4000 Da) caused a high degree of soft segment entanglement and formed many secondary bonds. This material exhibited better mechanical (tensile strength: 64.13 MPa and hardness: 90A) and thermomechanical properties than a bio-TPU based on poly(trimethylene succinate) glycol with Mn of 1000 Da. At an appropriate shape memory programming temperature, all synthesized bio-TPUs exhibited excellent shape memory behaviors with a fixed shape rate of more than 99% and a shape recovery rate of more than 86% in the first round and 95% in the following rounds. Authors confirmed the possibility of using bio-based poly(trimethylene succinate) glycols with different average molecular weights as potential polyols for obtaining shape-memory polyurethanes.

Our previous works involved a profound analysis regarding phase separation, energy activation, the value of melt flow index, and selected properties of bio-based thermoplastic polyurethanes synthesized via single and two different prepolymers (as their mixture) obtained with the usage of bio-based polyether polyols, poly(trimethylene glycol)s, with a wide range of average molecular weight [14,23,24]. Based on the obtained results, we noticed that it is possible to obtain TPU materials with expected properties via flexible phase controlling, which was our motivation for preparing this work.

In this study, green TPU materials were synthesized with the use of bio-based monomers, a prepolymer mixture, and a solvent-free method. The impacts of the chemical structure of flexible segments on selected properties of the prepared materials were investigated. The chemical structure of the prepared materials by spectroscopic and the following chromatographic techniques was investigated: Fourier transform infrared spectroscopy, proton nuclear magnetic resonance, and size-exclusion chromatography. Moreover, differential scanning calorimetry, thermogravimetric analysis, and dynamic mechanical analysis were used to investigate thermal and thermomechanical properties. Tensile strength and hardness were also determined. The relationship between the chemical structure and selected properties was described in detail.

## 2. Results and Discussion

### 2.1. Hydrogen Nuclear Magnetic Resonance Spectroscopy

**Figure 1:**
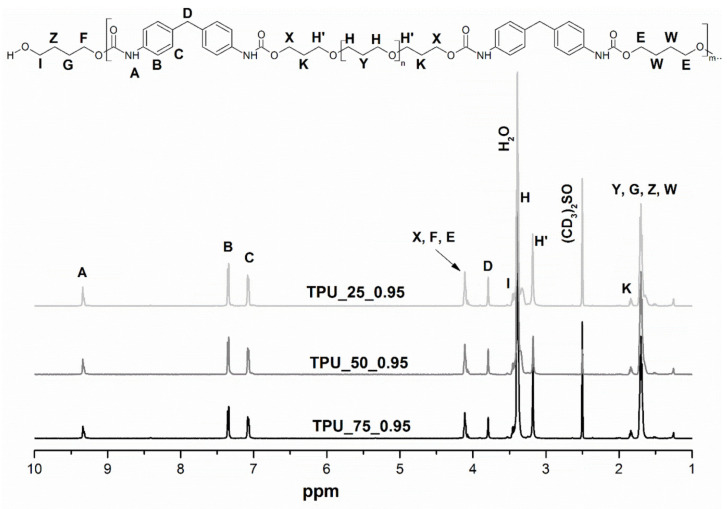
^1^H NMR spectra of prepared green TPUs.

Chemical compositions of thermoplastic polyurethanes were determined via ^1^H NMR. For illustration, 400 MHz ^1^H NMR spectra with a description of specific signals of the TPUs dissolved in DMSO-d_6_ (2.5 ppm) are demonstrated in Figure 1. The low-intensity signal at 9.35 ppm is assigned to the -NH in the urethane group. Two peaks appeared at 7.31 and 7.06 ppm, corresponding to the protons with two kinds of environment in the benzene ring, which indicates that the ring is substituted at the 1 and 4 positions. The CH_2_ located between the aromatic rings of MDI gives a characteristic signal at 3.77 ppm. The peaks at 1.70 and 3.17 ppm correspond to the CH_2_ of polyether diol and chain extender in the polymer chain. The signal at 4.09 ppm denotes that another kind of H in the main chain located next to the oxygen is also present in the structure of this type of material. On the basis of the analysis, it can be confirmed that the chain extender is OH-terminated diols. This is evidenced by the lack of peaks belonging to the urea appearing in the spectra [25].

### 2.2. Fourier Transform Infrared Spectroscopy

**Figure 2:**
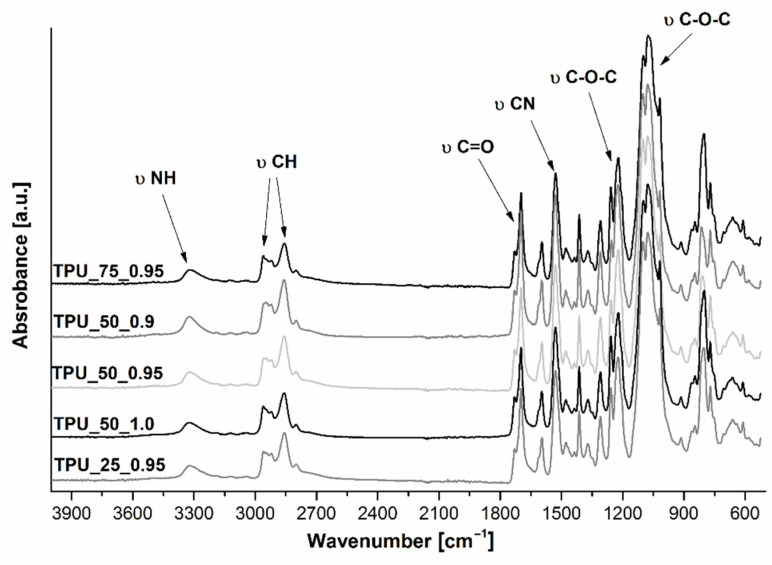
FTIR spectra of prepared green TPUs.

The chemical structure of TPUs was also examined via FTIR spectroscopy, and the spectra are presented in Figure 2. In general, all the obtained materials showed the presence of characteristic groups typical for polyurethanes and possessed similar chemical structures. The absorption bands assigned to the presence of -NCO (2270 cm^−1^) and -OH (3300–3500 cm^−1^) groups were not observed, suggesting that the polyaddition reaction between hydroxyl and isocyanate groups occurred completely. The peak with the maximum at 3325 cm^−1^ is assigned to the stretching vibration of -NH moieties in the urethane groups in the area of hydrogen bonds belonging to the hard segments. C-H asymmetric and symmetric stretching vibrations in the -CH_2_ groups were observed as bimodal bands with the maxima at 2858 and 2959 cm^−1^, respectively. The band at 1413 cm^−1^ is associated with the deformation vibrations of the C-H moieties. Moreover, the spectra showed a characteristic band between 1751 and 1640 cm^−1^ with two maxima, the bands at 1529 and 1310 cm^−1^, which are assigned to the stretching vibration of C=O, bending vibration of N-H in the -C-NH group, and stretching vibration of C-N, respectively [25]. It is worth noting that in the case of polyurethanes, the vibration band in the carbonyl region can be considered a sum of three peaks [24]. Typically, the bands range from 1640 to 1751 cm^−1^ and refer to the amide-I region. The absorbance at 1731 cm^−1^ is assigned to the free carbonyl group, whereas the peak at 1697 cm^−1^ is characteristic of the hydrogen-bonded C=O group in the crystalline phase of the hard segment domains [23]. Moreover, in the FTIR spectra of this type of material, the hydrogen-bonded C=O moiety stretching band in the amorphous phase was imperceptible [26]. The stretching vibrations of C-O-C in a polyether polyol chain could be coupled with the absorbance at 1080 cm^−1^. A characteristic band for the out-of-plane bending vibration of C-H in the 1,4-disubstituted aromatic ring is the absorbance at 817 cm^−1^. This peak can be coupled with the absorption band at 1596 cm^−1^, which is assigned to the skeletal vibration of C=C in the aromatic ring [27].

### 2.3. Size Exclusion Chromatography

**Figure 3:**
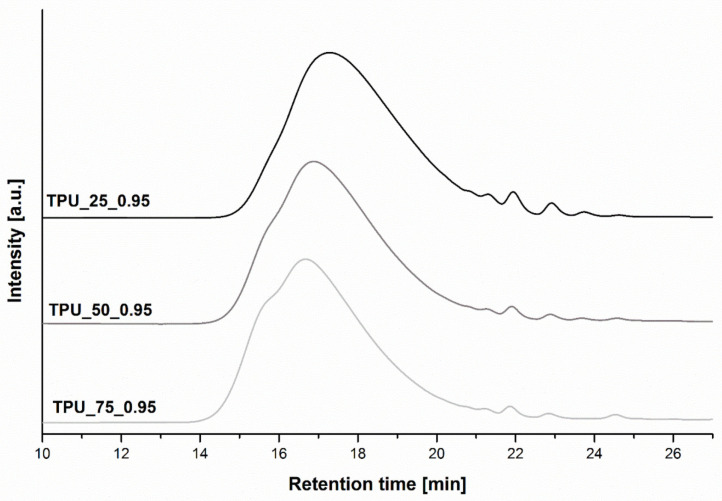
The GPC chromatograph of the prepared green TPUs.

The molecular weight distribution depended primarily on the molar ratio of the used components and the used catalyst, but also the reaction conditions such as the used temperature, pressure, and stirring, which had large impacts on the dispersity. Size-exclusion chromatography was used to characterize the number and weight average molecular weights and dispersity of the synthesized poly(ether-urethane)s. The impact of the different composition of the soft segments on the molecular structure is shown in Figure 3. Table 1 presents the statement of the SEC results. Distinct, broad peaks at similar retention times for all measured green thermoplastic polyurethane elastomers are visible on the graph. It is seen that with the higher amount of the poly(trimethylene glycol) with Mn at 2700 g/mol at the chemical structure of green TPUs, the synthesized TPUs revealed an extended retention time, from 16.72 min for the TPU_75_0.95 specimen to 17.30 min for the TPU_25_0.95 sample. It is well known that macromolecules with higher average molecular weights are characterized by a lower retention time. It is also seen in Table 2, in which increasing values of Mn and Mw are visible with decreasing retention time. The TPU based on 25 wt% of PO3G with an Mn of 1000 g/mol (TPU_25_0.95) was characterized by a lower value of Mn—ca. 20,000 g/mol and Mw ca. 40,000 g/mol. Other materials, with a higher amount of PO3G with Mn equaling 1000 g/mol at the soft segment structure, were characterized by higher values of Mn—ca. 28,000 g/mol and Mw ca. 50,000 g/mol for TPU_50_0.95 and ca. 55 000 g/mol for TPU_75_0.95, respectively. It is probably due to the higher mobility of the shorter chains that can react, compared to longer chains, whose mobility is hindered due to the chains’ length and their lower reactivity. Table 2 also shows the impact of the different [NCO]:[OH] functional groups’ molar ratio, used during the chain extender step, on the average molecular weight and dispersity of the green TPUs. The results allowed the confirmation that with higher [NCO]:[OH] molar ratio, the obtained TPUs are characterized by higher values of Mn and Mw. The highest value was exhibited by the sample TPU_50_1.0, Mn ca. 45,000 g/mol and Mw ca. 91,000 g/mol. It is known from the basic chemical reaction kinetics equations that with the use of a monomer molar ratio of 1, macromolecules characterized by the highest average molecular weight are synthesized. It can be expected that with increasing [NCO]:[OH] molar ratio, values of Mn and Mw will decrease.

### 2.4. Thermogravimetric Analysis

**Figure 4:**
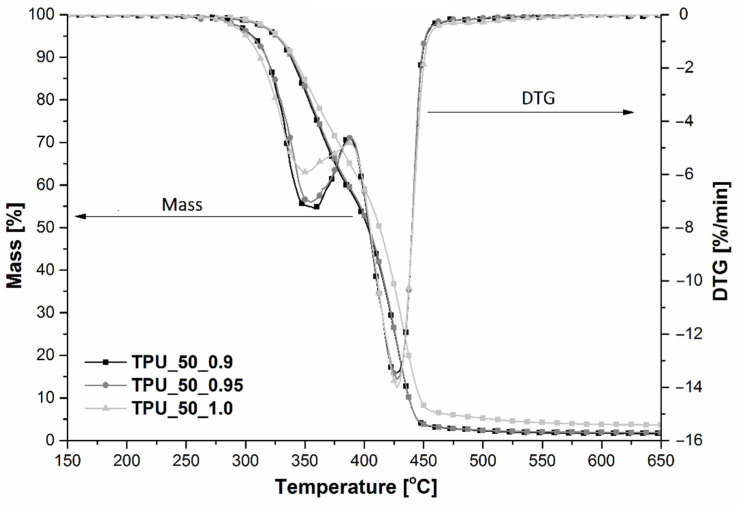
The dependence of mass/DTG and temperature for TPUs obtained in different [NCO]/[OH] molar ratios.

Thermal properties of polyurethanes have a large impact on their future application and mainly depend on the type of monomers used for their preparation, synthesis methods, and conditions or molar ratio of the main components [4]. The thermogravimetric measurements were performed in order to determine the beginning of thermal decomposition (T_5%_) of the prepared TPUs and the temperature at the maximum decomposition rate for each degradation step or char yield. For green TPUs synthesized in different [NCO]/[OH] molar ratios and by using a 1:1 mixture of two prepolymers, the thermograms of mass loss and DTG versus temperature were plotted, and they are shown in Figure 4, whereas the effect of different weight fractions of the two prepolymers on the thermal characteristic of TPUs is presented in Table 2.

In general, it can be observed that thermal degradation of each TPU took place in two independent stages regardless of the [NCO]/[OH] molar ratio or prepolymers contribution (Figure 4). Two-step thermal degradation typically occurred for segmented polyurethane materials and confirmed the good phase separation between flexible and hard segments [4,28,29]. At the first step, the thermal decomposition of hard segments (HS) composed of diisocyanate and bio-glycol residue was assigned, while at the second stage, the decomposition of flexible segments (SS) occurred. The changes in thermal behavior of TPUs indicate the beginning of the thermal decomposition (T_5%_), the maximum rate of each step (DTG_HS_ and DTG_SS_) of the thermal degradation, and its temperature (T_HS_ and T_SS_) and the amount of char yield (Table 2).

First, it was found that depending on the molar ratio, differences at the beginning of thermal degradation (T_5%_) occurred. It was noticed that with increasing [NCO]/[OH] molar ratio, T_5%_ increased, while with the increase in the amount of prepolymer based on a polyol with a lower molecular weight, a downward trend was observed in T_5%_. This is the effect of the higher amount of prepolymer based on the polyether polyol with a lower molecular weight (Mn = 1000 g/mol), and the same applies to the shorter molecular chain.

Next, analyzing the first step of the thermal decomposition, the visible effect of [NCO]/[OH] was recognized. With the increase in [NCO]/[OH] molar ratio, the thermal decomposition of the first step occurred at a lower temperature (up to 5 °C), while the speed of decomposition of HS decreased (see T_HS_ and DTG_HS_ given in Table 2). This is a result of the decrease in hard segments in the TPUs structure. Considering the contribution of each prepolymer in the TPUs, the same dependence was observed, which can be the effect of the lower molecular weight of the resulting TPU. An insignificant influence of the [NCO]/[OH] molar ratio and the prepolymer proportions on the maximum temperature at the second step of degradation (T_SS_) was noticed, whereas the decomposition speed, DTG_HS_, decreased with [NCO]/[OH] and with the prepolymer based on a polyol with a lower molecular weight.

### 2.5. Differential Scanning Calorimetry

**Figure 5:**
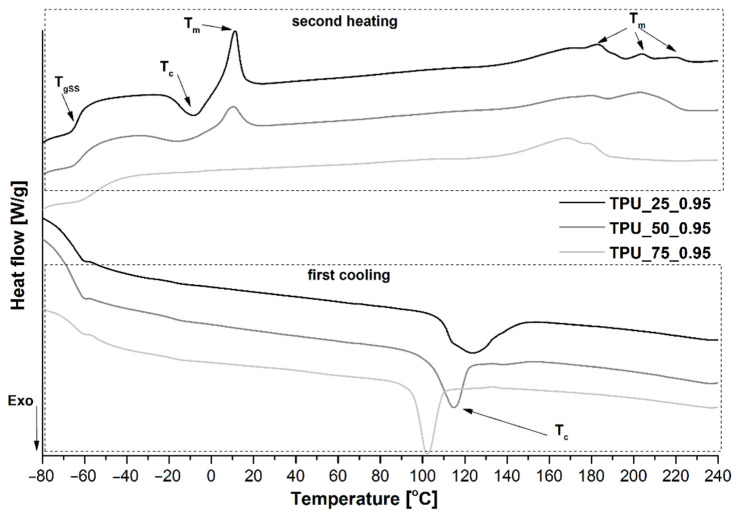
The endothermic (2nd run) and exothermic (1st run) curves of synthesized TPUs at the [NCO]/[OH] molar ratio 0.95.

DSC was used to investigate the thermal behavior of the obtained TPU and to confirm their segmented structure. The thermal effects such as the glass transition of flexible segments, melting, and crystallization (including cold crystallization) were noticed and are presented as endothermic and exothermic curves in Figure 5, while all determined values of the mentioned parameters are listed in Table 3. The influence of each prepolymer contribution that was used for TPU synthesis was revealed in the glass temperature of flexible segments and in melting temperature of SS and HS. Increasing the prepolymer content based on polyol PO3G2700 led to a decrease in T_gSS_ and favored the cold crystallization of SS because of the higher amount of the methylene sequences in the structure of the PO3G2700 polyol and the same mobility of the polymer chains [30]. The sample TPU_25_0.95 revealed the highest total ΔH_c_ and a high ΔH_m_, which suggests that these materials are characterized by the highest degree of phase separation [24]. With increasing prepolymer contribution based on the PO3G1000 polyol, this effect disappeared and, in consequence, in the case of TPU_75_0.95, the melting temperature of SS was also invisible on the endothermic curve. Analyzing the hard segments, it is impossible to determine the glass transition temperature T_gHS_, which suggests the lack of well-ordered hard domains in hard segments. The melting of HS was registered as broad multiple peaks. Depending on the prepolymer fractions, the melting peaks are characterized by two (samples: TPU_50_0.95 and TPU_75_0.95) or three maxima (TPU_25_0.95). This finding indicates a disordering in the hard segments, and partial miscibility of the hard phase in soft segments, which is also confirmed by a decrease in the enthalpy of crystallization. The difference in the behavior of the melting of TPU materials suggests that they have different physical origins. The first melting peak at the lowest temperature was assigned to the ordered structure present in the hard phase, and peaks at the higher temperature were related with phase mixing of SS and HS [31].

### 2.6. Dynamic Mechanical and Thermal Analysis

**Figure 6:**
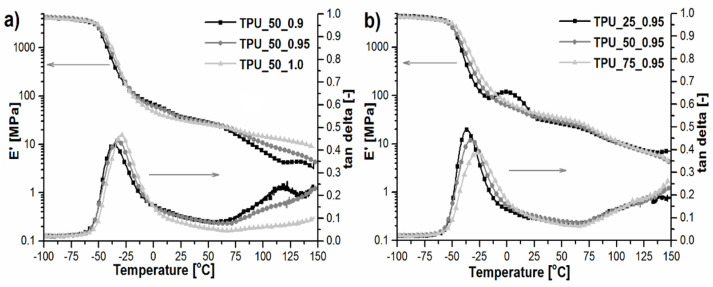
The storage modulus (E’) and tangent delta (tan δ) as a function of temperature for TPUs synthesized by using a different [NCO]/[OH] molar ratios and by using an equal amount of both prepolymers (based on PO3G1000 and PO3G2700) (**a**), and for TPUs synthesized by using the same [NCO]/[OH] molar ratio and (**b**) different amounts of both prepolymers (based on PO3G1000 and PO3G2700).

Dynamic mechanical analysis supports the thermal analysis of TPUs in the aspect of their viscoelastic behavior. Figure 6 shows the storage modulus (E’) and tangent delta (tan δ) dependence of temperature for TPUs synthesized by using a different [NCO]/[OH] molar ratio and by using an equal amount of both prepolymers (based on PO3G1000 and PO3G2700) (a), and for TPUs synthesized by using the same [NCO]/[OH] molar ratio and different amounts of both prepolymers (based on PO3G1000 and PO3G2700) (b).

Regardless of the [NCO]/[OH] molar ratio or the two types of the prepolymer contribution in the synthesized TPUs, the storage modulus in the glassy state and at room temperature exhibited almost the same values, slightly higher for TPU_75_0.95, which indicated the presence of a hard phase in flexible segments. The changes caused by differences in flexible segments were noticed as the α transition temperature. With an increasing [NCO]/[OH] molar ratio and the amount of prepolymers based on polyol with a lower molecular weight, the T_gSS_ increased, which is related to the lower mobility of the polymer chain. Above the T_gSS_ for the sample coded TPU_25_0.95, the effect of cold crystallization was revealed (in agreement with DSC results) as a peak on E’ curves. Above the 75 °C, the transition of hard segments was visible (Figure 6), especially in the case of TPU_50_0.9 where a second plateau might be well observed. This material was characterized by the highest melting temperature, determined based on DSC measurements. All TPU materials exhibit the tendency to melt above T_gSH_. Based on tan delta curves, not only were the temperatures of phase transitions analyzed, but also the damping properties. The [NCO]/[OH] molar ratio did not significantly affect the damping coefficient. The mentioned parameter depends on the proportion of two types of prepolymers differing in soft segment molecular weight, which were used in the TPUs synthesis. Increasing the contribution of short soft segments caused a decrease in the polymer chain mobility, which, in turn, caused a decrease in damping. Similar findings were described in our previous works [23,24].

Table 5 presents the collection of DMA data obtained from tan δ versus temperature curves courses. The results indicate that with an increase in the amount of prepolymer based on PO3G1000-polyol and with the increase in [NCO]/[OH] molar ratio, the Tg also increased, apart from TPU_25_0.95. With the increase in the amount of methylene groups–CH_2_-, at the chemical structure of the polyol, the values of Tg shifted toward lower values. Moreover, with the increase in [NCO]/[OH] molar ratio, there may be a greater proportion of hydrogen bonds, which translates into a stiffer material and higher Tg values. The damping temperature range is defined as the temperature in which tan δ > 0.3 [8]. The presented results showed that the highest value of the temperature range for efficient damping characterized the green TPU material coded TPU_50_1.0, where the equal mass quantity of both prepolymers was used. It is directly related to the value of the average molecular weight of this material, which was confirmed by GPC measurements (Table 2). With increasing macromolecular chain length, the materials were characterized by better damping ability. Moreover, it is visible that with the increase in the [NCO]/[OH] molar ratio, the damping temperature range also increased. The value of the integral area from −60 to 62.5 °C in the tan δ versus temperature curves can be defined as an additional supplementary indicator of damping performance. As shown in Table 4, all prepared green TPUs revealed a great efficient energy dissipation, which endowed them with the potential as a green material with high damping capacity.

### 2.7. The Mechanical Properties

The chemical structure of macromolecules has a large impact on the physicochemical, thermal, and mechanical properties of polyurethanes. Due to their segmented structure, TPU materials are characterized by their specific elastomeric properties. Long chains of soft segments give the materials high values of elongation but lower tensile strength than TPUs based on soft segments formed by shorter chains. Mechanical properties such as tensile strength (TS_b_), elongation at break (ε_b_), and hardness (H) of the prepared green TPUs were measured, and the results are shown in Table 5. Clearly visible is the fact that with an increasing amount of poly(trimethylene glycol) with Mn at ca. 1000 g/mol at the soft segment structure, the tensile strength of the green TPUs also increased. The value of TS_b_ for TPU_25_0.95 equaled 13.3 MPa, whereas the sample TPU_75_0.95 exhibited TS_b_ at 24.5 MPa. Nevertheless, a higher amount of lower-average-molecular-mass poly(trimethylene glycol) at the soft segment structure caused a decrease in elongation at break. Samples with a higher amount of PO3G2700 than PO3G1000 at the soft segment structure characterized the higher elongation. The highest value ca. 570% was obtained for TPU_25_0.95 and the lowest—TPU_75_0.95 (ca. 398%). The molar ratio of [NCO]/[OH] groups also influenced the tensile strength and elongation at break of the TPU materials. The results indicate that with the increase in [NCO]/[OH] molar ratio, the tensile strength of the materials also increased. The elongation at break results did not reveal a growing trend.

The results of hardness measurements confirmed that an increasing amount of PO3G with 1000 g/mol at the soft segment structure led to TPUs with a higher hardness. The same trend was also visible in the case of increasing [NCO]/[OH] molar ratio.

### 2.8. Melt Flow Index

The melt flow index value provides important information on assessing the processability of polymeric materials. It was observed that both the molar ratio and the proportion of the used prepolymers affected the MFR and MVR values (Table 6), which was expected. Therefore, the MFR values gradually decreased with [NCO]/[OH] molar ratio during the prepolymer chain extending step, and it was connected to the hard segment concentration in the chemical structure. It was also observed that the addition of a prepolymer synthesized from PO3G1000 resulted in a significant increase in the MFR value, which is favorable in terms of reprocessing.

## 3. Materials and Methods

### 3.1. Materials

- The bio-based poly(trimethylene glycol) (PO3G 1000) (Mn = 1000 g/mol, hydroxyl number 125–102 mgKOH/g, acid value <0.05 mgKOH/g, viscosity (at 40 °C) 200–300 mPa·s) was provided by Allesa (Frankfurt, Germany).
- The bio-based poly(trimethylene glycol) (PO3G 2700) (Mn = 2700 g/mol, hydroxyl number 43.2–40.1 mgKOH/g, acid value <0.05 mgKOH/g, viscosity (at 40 °C) 1450–1850 mPa·s) was provided by Allesa (Frankfurt, Germany).
- 4,4′-diphenylmethane diisocyanate (MDI) (NCO content: 33.5%, purity 99.5%) was purchased from Borsod Chem (Budapest, Hungary).
- The bio-based glycols: 1,4-butanediol (bio-BDO) (purity 99.8%)
- The catalyst 1,4-diacabicyclo [2.2.2]octane (DABCO) was purchased from Sigma-Aldrich (Warsaw, Poland).

### 3.2. Preparation of Thermoplastic Polyurethane Elastomers

**Figure 7:**
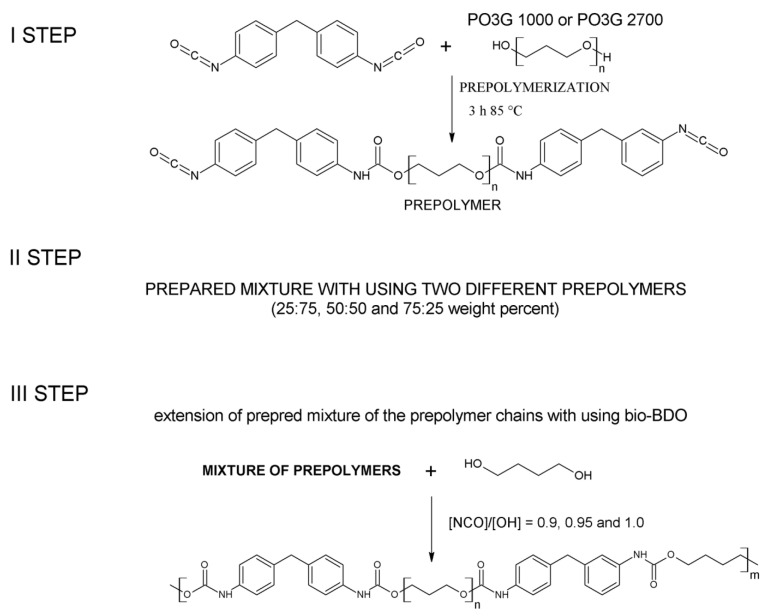
The schematic synthesis of green TPUs obtained by using the mixture of prepolymers.

Green TPUs based on poly(trimethylene glycol) with molecular weights of 1000 and 2700 g/mol were synthesized using a two-step method [2,23,24]. The scheme of the prepolymer preparation is presented in Figure 7. The percentage of the unreacted NCO groups in both prepolymers equaled 7%, which was determined according to the ISO 14896:2010 standard. The ratios of both different prepolymers based on the two mentioned bio-based polyols with different Mn values were determined as 25:75, 50:50, and 75:25 wt.%. Bio-based 1,4-butanediol was used as a chain extender. DABCO was used as a catalyst in the solution at bio-BDO with a 0.3 wt.% concentration. Green TPUs were obtained at three [NCO]/[OH] molar ratios—0.9, 0.95, and 1.0. The content of hard and soft segments, formulation, molar ratio of [NCO]/[OH], and sample codes that have been used throughout the work has been presented in Table 7.

The first number in the symbol corresponds to the content of prepolymer based on PO3G1000, while the second number determines the [NCO]/[OH] molar ratio. For instance, the material marked as TPU_25_0.95 was obtained from the prepolymer mixture that contains 25 wt.% of prepolymer synthesized from polyol with a molecular weight equal to 1000 g/mol and 75 wt.% of prepolymer based on polyol with a molecular weight equal to 2700 g/mol, at the [NCO]/[OH] molar ratio of 0.95.

### 3.3. Characterization of Thermoplastic Polyurethane Elastomers

Hydrogen Nuclear Magnetic Resonance Spectroscopy (^1^H NMR) Was Carried Out by using the Varian Mercury Vx Spectrometer. Samples Dissolved in DMSO-d6 Were Measured at Room Temperature with the Operating Frequency of the Spectrometer at 400 MHz.

Fourier Transform Infrared Spectroscopy (FTIR) Was Carried Out Using a Nicolet 8700 FTIR Spectrophotometer (Thermo Electron Co., USA) in Attenuated Total Reflection (ATR) Mode. The Measurements Were Carried out at Room Temperature for Wavenumbers Ranging from 500 to 4000 cm^−1^ with a Resolution Equal to 4 cm^−1^ and 64 Scans.

Size Exclusion Chromatography (SEC) Was Performed to Determine the Number (Mn) and Weight Average (Mw) Molecular Weights and Dispersity. A Chromatographic System Equipped with a Refractive Index Detector (Shodex, Japan), UV-Vis Detector (λ = 254 nm, LCD 2084, Ecom, Czech Republic), and a Set of Three Columns (PLgel with a Particle Size of 10 μm, Pore Size: 50/10E3/10E4 Å, 300 × 7.5 mm, Polymer Laboratories, UK) Was Used. Polystyrene Standards were Employed for Calibration and Tetrahydrofuran was Applied as the Eluent.

Thermogravimetric Analysis (TGA) Was Performed Using a NETZSCH TG 209 Libra. The Analyses Were Performed under Nitrogen Flow within the Temperature Range of 35–700 °C and at a Heating Rate of 10 °C min^−1^.

Differential Scanning Calorimetry (DSC) Was Performed Using a Q 2000 Calorimeter (TA Instruments). The Measurements Were Carried Out in a Heating-Cooling-Heating Cycle at the Temperature Range of 90–250 °C. The Heating Rate was 10 °C/min and a Nitrogen Atmosphere was Employed. The Sample Weight was Approximately 6 mg.

Dynamic Mechanical and Thermal Analysis (DMTA) Was Carried out Using the DMA Q800 Analyzer (TA Instruments) Following ASTM D4065:2012. The Measurement Temperature Ranged from −100 to 150 °C, the Heating Rate Equaled 4 °C/min, and the Frequency Was 10 Hz.

The Mechanical Properties under Static Conditions Were Investigated on a Zwick Z020 Tensile Testing Machine, According to the ISO 37:2007 Standard. The Dumbbell Specimens Were Stretched at a Cross-head Speed of 100 mm/min, at Room Temperature. The Hardness Measurements Were Carried out Using an Electronic Shore Type A Durometer, Following the ISO 868:2005 Standard. The Results were Determined as the Average of Ten Measurements.

The Melt Flow index (MFI) of Green Thermoplastic Polyurethane Elastomers Were Measured Using a Zwick/Roell Plastometer, under ISO 1133. The Evaluations Were Conducted with a 5.0 kg Load at 175 °C.

## 4. Conclusions

The chemical structure of the synthesized green TPUs depends on both the amount of each type of different prepolymer (differs in molecular weight of flexible segments) and the [NCO]/[OH] molar ratio. Material prepared with the use of an equilibrium amount of prepolymers and an equimolar concentration of [NCO]/[OH] groups showed the highest value of number average molecular weight (ca. 45,000 Da), which resulted from the highest [NCO]/[OH] molar ratio. All TPUs exhibit a segmented structure, which impacts the thermal properties, analyzed via DSC, TG, and DMA techniques. The increasing amount of prepolymers based on polyol with a lower molecular weight decreased the total crystallization and melting enthalpy, ΔHc and ΔHm, respectively. The same trend was also observed in the case of [NCO]/[OH] molar ratio, where with increasing molar ratio, ΔHc and ΔHm decreased, which is directly related to the degree of phase separation. TG analysis confirmed the high thermal stability of all green TPUs. All materials characterized the beginning of thermal degradation temperature at ca. 350 °C. Moreover, based on DMA results, it was confirmed that the prepared TPUs revealed good damping capacity at a broad temperature range, which is the effect of two macromolecular mass-differed polyols usage. Based on mechanical tests results, it was confirmed that both increasing the [NCO]/[OH] molar ratio and amount of PO3G1000 increased the tensile strength and hardness of the prepared materials. The highest value of the elongation at break characterized sample TPU_25_0.95 (ca. 570%), which is directly related with the degree of phase separation. The value of melt flow index depended mainly on the [NCO]/[OH] molar ration, which is related with the content of the hard segments. It can be stated that each of the TPUs might be processed by using various methods typical for thermoplastic materials, such as 3D printing, injection molding, or extrusion. The obtained results confirmed the possibility to design selected properties of green thermoplastic polyurethane elastomers based on their required usage features. Due to that, we can promote a new type of bio-based TPU as a base for the production of everyday life items.

## Figures and Tables

**Table 1:** The results of the GPC measurements.

Sample	[NCO]/[OH]	M_n_	M_w_	Đ	Retention Time of the Peak (min)
TPU_25_0.95	0.95	19,742	39,485	2.0	17.30
TPU_50_0.9	0.9	22,075	41,944	1.9	17.02
TPU_50_0.95	0.95	27,535	49,563	1.8	16.88
TPU_50_1.0	1.0	45,342	90,684	2.0	15.58
TPU_75_0.95	0.95	28,866	54,846	1.9	16.72

M_n_—number average molecular weight; M_w_—weight average molecular weight, Đ—dispersity.

**Table 2:** Thermal characteristic of synthesized thermoplastic polyurethanes based on TG measurements.

Sample	T_5%_ [°C]	T_10%_ [°C]	T_90%_ [°C]	Char Yield [%]	T_HS_ [°C]	DTG_HS_ [%/min]	T_SS_ [°C]	DTG_SS_ [%/min]
TPU_25_0.95	332.1	342.1	441.2	1.77	362.8	−6.5	426.2	−15.4
TPU_50_0.9	324.1	337.4	436.8	1.35	357.5	−7.2	427.5	−13.4
TPU_50_0.95	326.0	338.9	436.9	1.89	354.9	−7.0	427.4	−13.7
TPU_50_1.0	327.3	339.2	442.1	3.42	349.9	−5.9	427.4	−14.0
TPU_75_0.95	317.2	336.2	429.3	4.21	351.8	−10.4	429.8	−8.2

**Table 3:** Thermal properties of synthesized TPUs determined by DSC technique.

Sample	T_gSS_ (°C)	T_c_ (°C)	ΔH_c_ (J/g)	Total ΔH_c_ (J/g)	T_m_ (°C)	ΔH_m_ (J/g)	Total ΔH_m_ (J/g)
TPU_25_0.95	−63.7	−8.0 123.2	3.52 9.12	12.64	11.30 170.4 182.5 203.2 218.7	3.47 9.05	12.52
TPU_50_0.9	−62.2	−16.8 121.3	4.21 8.42	12.63	8.6 176.2 198.6	3.01 9.82	12.83
TPU_50_0.95	−61.3	−14.1 116.5	2.10 8.29	10.39	10.4 180.7 202.9	2.22 8.53	10.75
TPU_50_1.0	−58.6	110.2	7.21	7.21	10.1 184.5	0.97 6.34	7.31
TPU_75_0.95	−54.9	101.8	8.90	8.90	168.7	8.57	8.57

ΔHc—crystallization enthalpy, Tc—crystallization temperature, Tm—melting temperature, TgSS—glass transition temperature of the soft segments, ΔHm—melting enthalpy.

**Table 4:** DMA results of the prepared green TPUs.

Sample	T_gSS_ [°C]	tan δ_max_	ΔT [°C]	S_−60–62.5 °C_
TPU_25_0.95	−35.8	0.49	23.3	15.6
TPU_50_0.9	−36.0	0.42	23.5	14.6
TPU_50_0.95	−32.0	0.44	24.6	16.3
TPU_50_1.0	−29.0	0.47	25.4	15.7
TPU_75_0.95	−27.0	0.39	23.5	15.2

△T—the temperature range for efficient damping (tan δ > 0.3); S_−60−62.5 °C_—the integral area from −60 °C to 62.5 °C in tan δ temperature curves.

**Table 5:** The results of selected mechanical properties of the prepared green TPUs.

Sample	TS_b_ [MPa]	ε_b_ [%]	H [^°^ShD]
TPU_25_0.95	13.3 ± 0.8	570 ± 12	23.1 ± 0.7
TPU_50_0.9	8.2 ± 0.8	228 ± 13	23.1 ± 0.2
TPU_50_0.95	17.5 ± 1.3	460 ± 21	27.5 ± 1.1
TPU_50_1.0	24.7 ± 0.9	430 ± 19	29.8 ± 0.9
TPU_75_0.95	24.5 ± 1.2	398 ± 10	32.3 ± 0.8

**Table 6:** The results of selected mechanical properties of the prepared green TPUs.

Sample	MFR [g/10 min]	MVR [cm^3^/10 min]
TPU_25_0.95	14.4 ± 0.3	13.8 ± 0.2
TPU_50_0.9	37.2 ± 0.4	36.8 ± 0.3
TPU_50_0.95	22.1 ± 0.2	21.8 ± 0.3
TPU_50_1.0	17.5 ± 0.3	17.2 ± 0.1
TPU_75_0.95	33.7 ± 0.2	33.1 ± 0.1

MFR—melt mass-flow rate, which is the mass flow expressed in g/10 min; MVR—melt volume-flow rate, which is the volume flow expressed in cm^3^/10 min.

**Table 7:** Codes and formulation (molar ratio [NCO]/[OH], hard segments (HS), and soft segments (SS)) of synthesized green TPUs.

Sample	PO3G 1000 [wt.%]	PO3G 2700 [wt.%]	[NCO]/[OH]	Monomers Molar Ratio PO3G1000:PO3G2700:MDI:BDO	HS [wt.%] *	SS [wt.%] *	%BIO_BASED CONTENT **
TPU_25_0.95	25	75	0.95	1:1.25:7.44:2.50	32.27	67.73	71.18
TPU_50_0.9	50	50	0.9	1:0.41:4.00:1.31	34.60	65.40	69.04
TPU_50_0.95	50	50	0.95	1:0.41:4.00:1.25	34.47	65.53	68.98
TPU_50_1.0	50	50	1.0	1:0.41:4.00:1.19	34.36	65.64	68.92
TPU_75_0.95	75	25	0.95	1:0.15:2.94:0.85	36.68	63.32	66.77

* HS and SS were calculated on the basis of Kasprzyk et al. (Kasprzyk and Datta, 2019); ** determined on the basis of the formula presented by Kasprzyk et al. [2].

## Data Availability

Data are contained within the article. The data presented in this study are available in Green TPUs from prepolymer mixtures designed by controlling the chemical structure of flexible segments.

## References

[1] Datta J., Kasprzyk P. (2017). Thermoplastic Polyurethanes Derived From Petrochemical or Renewable Resources: A Comprehensive Review. Polym. Eng. Sci..

[2] Kasprzyk P., Datta J. (2019). Novel Bio-Based Thermoplastic Poly(Ether-Urethane)s. Correlations between the Structure, Processing and Properties. Polymer.

[3] Petrović Z.S., Milić J., Zhang F., Ilavsky J. (2017). Fast-Responding Bio-Based Shape Memory Thermoplastic Polyurethanes. Polymer.

[4] Parcheta P., Głowińska E., Datta J. (2020). Effect of Bio-Based Components on the Chemical Structure, Thermal Stability and Mechanical Properties of Green Thermoplastic Polyurethane Elastomers. Eur. Polym. J..

[5] Guo Y., Zhang R., Xiao Q., Guo H., Wang Z., Li X., Chen J., Zhu J. (2018). Asynchronous Fracture of Hierarchical Microstructures in Hard Domain of Thermoplastic Polyurethane Elastomer: Effect of Chain Extender. Polymer.

[6] Eceiza A., Martin M.D., De La Caba K., Kortaberria G., Gabilondo N., Corcuera M.A., Mondragon I. (2008). Thermoplastic Polyurethane Elastomers Based on Polycarbonate Diols With Different Soft Segment Molecular Weight and Chemical Structure: Mechanical and Thermal Properties. Polym. Eng. Sci..

[7] Powers D.S., Vaia R.A., Koerner H., Serres J., Mirau P.A. (2008). NMR Characterization of Low Hard Segment Thermoplastic Polyurethane/Carbon Nanofiber Composites. Macromolecules.

[8] Zhao X., Shou T., Liang R., Hu S., Yu P., Zhang L. (2020). Bio-Based Thermoplastic Polyurethane Derived from Polylactic Acid with High-Damping Performance. Ind. Crop. Prod..

[9] Błażek K., Datta J. (2019). Renewable Natural Resources as Green Alternative Substrates to Obtain Bio-Based Non-Isocyanate Polyurethanes-Review. Crit. Rev. Environ. Sci. Technol..

[10] Parcheta P., Datta J. (2017). Environmental Impact and Industrial Development of Biorenewable Resources for Polyurethanes. Crit. Rev. Environ. Sci. Technol..

[11] Głowińska E., Kasprzyk P., Datta J. (2021). The Green Approach to the Synthesis of Bio-Based Thermoplastic Polyurethane Elastomers with Partially Bio-Based Hard Blocks. Materials.

[12] Głowińska E., Wolak W., Datta J. (2021). Eco-Friendly Route for Thermoplastic Polyurethane Elastomers with Bio-Based Hard Segments Composed of Bio-Glycol and Mixtures of Aromatic–Aliphatic and Aliphatic–Aliphatic Diisocyanate. J. Polym. Environ..

[13] Głowińska E., Parcheta P., Kasprzyk P., Janusz D. (2021). Book chapter: Polyisocyanates from Sustainable Resources. Polyurethane Chemistry: Renewable Polyols and Isocyanates.

[14] Kasprzyk P., Datta J. (2018). Effect of Molar Ratio [NCO]/[OH] Groups during Prepolymer Chains Extending Step on the Morphology and Selected Mechanical Properties of Final Bio-Based Thermoplastic Poly ( Ether-Urethane ) Materials. Polym. Eng. Sci..

[15] Zeng B., Li Y., Wang L., Zheng Y., Shen J., Guo S. (2020). Body Temperature-Triggered Shape-Memory Effect via Toughening Sustainable Poly(Propylene Carbonate) with Thermoplastic Polyurethane: Toward Potential Application of Biomedical Stents. ACS Sustain. Chem. Eng..

[16] Blache H., Méchin F., Rousseau A., Fleury É., Pascault J.P., Alcouffe P., Jacquel N., Saint-Loup R. (2018). New Bio-Based Thermoplastic Polyurethane Elastomers from Isosorbide and Rapeseed Oil Derivatives. Ind. Crop. Prod..

[17] Sonnenschein M.F., Ginzburg V.V., Schiller K.S., Wendt B.L. (2013). Design, Polymerization, and Properties of High Performance Thermoplastic Polyurethane Elastomers from Seed-Oil Derived Soft Segments. Polymer.

[18] Datta J., Głowińska E. (2014). Effect of Hydroxylated Soybean Oil and Bio-Based Propanediol on the Structure and Thermal Properties of Synthesized Bio-Polyurethanes. Ind. Crop. Prod..

[19] Ghosal K., Bhattacharjee U., Sarkar K. (2019). Facile Green Synthesis of Bioresorbable Polyester from Soybean Oil and Recycled Plastic Waste for Osteochondral Tissue Regeneration. Eur. Polym. J..

[20] Tawade B.V., Shingte R.D., Kuhire S., Sadavarte N., Garg K., Maher D., Ichake A., More A.S., Wadgaonkar P.P. (2017). Bio-Based Di/ Polyisocyanates for Polyurethanes: An Overview. Polyurethanes Today.

[21] https://www.weylchem.com.

[22] Pattamaprom C., Wu C.H., Chen P.H., Huang Y.L., Ranganathan P., Rwei S.P., Chuan F.S. (2020). Solvent-Free One-Shot Synthesis of Thermoplastic Polyurethane Based on Bio-Poly(1,3-Propylene Succinate) Glycol with Temperature-Sensitive Shape Memory Behavior. ACS Omega.

[23] Kasprzyk P., Sadowska E., Datta J. (2019). Investigation of Thermoplastic Polyurethanes Synthesized via Two Different Prepolymers. J. Polym. Environ..

[24] Kasprzyk P., Benes H., Keitel R., Datta J. (2020). The Role of Hydrogen Bonding on Tuning Hard-Soft Segments in Bio- Based Thermoplastic Poly (Ether-Urethane) s. J. Clean. Prod..

[25] Tang Q., Gao K. (2017). Structure Analysis of Polyether-Based Thermoplastic Polyurethane Elastomers by FTIR, 1H NMR and 13C NMR. Int. J. Polym. Anal. Charact..

[26] Saralegi A., Rueda L., Fernández-D’Arlas B., Mondragon I., Eceiza A., Corcuera M.A. (2013). Thermoplastic Polyurethanes from Renewable Resources: Effect of Soft Segment Chemical Structure and Molecular Weight on Morphology and Final Properties. Polym. Int..

[27] Rosu D., Rosu L., Cascaval C.N. (2009). IR-Change and Yellowing of Polyurethane as a Result of UV Irradiation. Polym. Degrad. Stab..

[28] Głowińska E., Datta J., Rodríguez Romero J.F., Simón Herrero D., Carmona M. (2018). Novel Cast Polyetherurethanes Based on Dispersed Polymeric Polyols. Polym. Test..

[29] Głowińska E., Datta J., Parcheta P. (2017). Effect of Sisal Fiber Filler on Thermal Properties of Bio-Based Polyurethane Composites. J. Therm. Anal. Calorim..

[30] Zajac M., Kahl H., Schade B., Rödel T., Dionisio M., Beiner M. (2017). Relaxation Behavior of Polyurethane Networks with Different Composition and Crosslinking Density. Polymer.

[31] Saiani A., Novak A., Rodier L., Eeckhaut G., Leenslag J.W., Higgins J.S. (2007). Origin of Multiple Melting Endotherms in a High Hard Block Content Polyurethane. 1. Thermodynamic Investigation. Macromolecules.

